# Clinical and Histological Prognostic Factors of Recurrence and Malignant Transformation in a Large Series of Oral Potentially Malignant Disorders

**DOI:** 10.3389/fonc.2022.886404

**Published:** 2022-04-21

**Authors:** Luigi Lorini, Michele Tomasoni, Cristina Gurizzan, Chiara Magri, Mattia Facchetti, Simonetta Battocchio, Chiara Romani, Marco Ravanelli, Arianna Oberti, Anna Bozzola, Elena Bardellini, Alberto Paderno, Davide Mattavelli, Davide Lombardi, Alberto Grammatica, Alberto Deganello, Fabio Facchetti, Stefano Calza, Alessandra Majorana, Cesare Piazza, Paolo Bossi

**Affiliations:** ^1^ Medical Oncology Unit, Department of Medical and Surgical Specialties, Radiological Sciences and Public Health, University of Brescia, ASST Spedali Civili, Brescia, Italy; ^2^ Otorhinolaryngology-Head and Neck Surgery Unit, Department of Medical and Surgical Specialties, Radiological Sciences and Public Health, University of Brescia, ASST Spedali Civili, Brescia, Italy; ^3^ Department of Pathology, ASST Spedali Civili, Brescia, Italy; ^4^ Angelo Nocivelli Institute of Molecular Medicine, University of Brescia and ASST Spedali Civili, Brescia, Italy; ^5^ Department or Radiology, University of Brescia, ASST Spedali Civili;, Brescia, Italy; ^6^ Dental Clinic, Oral Medicine Unit, Department of Medical and Surgical Specialties, Radiological Sciences and Public Health, University of Brescia, ASST Spedali Civili, Brescia, Italy; ^7^ Biostatistics and Bioinformatics Unit, Department of Molecular and Translational Medicine, University of Brescia, Brescia, Italy; ^8^ BDbiomed, Big and Open Data Innovation Laboratory, University of Brescia, Brescia, Italy

**Keywords:** oral potentially malignant disease, oral squamous cell carcinoma (OSCC), head and neck squamous cell carcinoma (HNSCC), oral carcinoma risk factors, prevention of malignant transformation

## Abstract

**Background:**

Oral potentially malignant disorders (OPMDs) represent a heterogeneous set of different histological lesions, characterized by the capacity to transform in oral squamous cell carcinoma (OSCC). Despite optimal surgical treatment, approximately 20%–30% of OPMDs may evolve into OSCC. No clear clinical/histological factors are able to identify OPMDs at higher risk of malignant transformation.

**Materials and Methods:**

We considered surgically treated patients with a diagnosis of OPMDs, enrolled from 1996 to 2019 at ASST Spedali Civili of Brescia without a diagnosis of OSCC within the previous 2 years. Clinical and histological characteristics were recorded. Outcomes of interest were recurrence-free survival (RFS), defined as the time from surgery for primary OPMD to any relapse of OPMD or malignant transformation, whichever occurred first, and carcinoma-free survival (CFS), defined as the time from surgery for OPMD to malignant transformation.

**Results:**

We retrospectively reviewed 106 OPMDs cases. Median age at first diagnosis was 64 years old (IQR = 18.75); female patients comprise 51.9% of the cases. During a median follow-up of 30.5 months (IQR = 44), in 23.5% of patients, malignant transformation occurred. RFS at 1, 5, and 10 years was 92.4%, 60.9%, and 43.2%, respectively. Female sex and history of previous OSCC were independent risk factors for RFS. CFS at 1, 5, and 10 years of follow-up was 97.1%, 75.9%, and 64.4%, respectively. Previous OSCC was an independent risk factor for CFS.

**Conclusions:**

In this large series of OPMDs, only previous diagnosis of OSCC was a prognostic factor for further OSCC occurrence. Given the lack of additional clinical/pathological prognostic factors, we advocate further studies into molecular characterization of OPMDs to better stratify the risk of malignant transformation.

## Introduction

More than 90% of oral cancers are represented by OSCCs. OSCCs are among the most frequent neoplasms worldwide, with an increasing incidence, especially in developing countries (1). Prognosis of oral cancer is poor, with an overall 5-year overall survival (5-year OS) ranging from 40%, if diagnosed in advanced stage (III–IV), to 80% if diagnosed in early stage (I–II) (2). Unfortunately, more than half of oral cancers are diagnosed when already in stage III–IV (3). Given the high mortality rate of advanced oral cancers, their early detection and anticipation in diagnosis can result in a significant gain in patient survival (4). Oral potentially malignant disorders (OPMDs), a group of oral mucosal lesions at increased risk of malignant transformation, represent the most common oral precancerous condition with a prevalence worldwide of approximately 4.5% (5). OPMDs comprise various entities such as leukoplakia, erythroplakia, erythroleukoplakia, proliferative verrucous leukoplakia, oral lichen planus, oral submucous fibrosis, oral lichenoid lesion, oral graft versus host disease, and oral dysplasia (6, 7). Currently, the staging proposed by the World Health Organization (WHO) is the most widely accepted, dividing OPMDs based on the grade of dysplasia (low, moderate, high/carcinoma *in situ*) and assigning them a different risk of malignant transformation (6%, 18%, and 39%, respectively) (8). The main risk factors for the development of OPMDs are shared with OSCC and are represented by smoking and alcoholic abuse, derivates of betel nut and areca nut, human papillomavirus infection (HPV) mainly type 16, oral mucosal trauma, chronic mucosal inflammation, and genetic diseases such as the Fanconi Anemia or Xeroderma pigmentosum (9). None of these risk factors, generally considered in the etiopathogenesis of OPMDs, have consistent data, across studies, as a prognosticator of malignant transformation. Identification of patients with OPMDs at higher risk of malignant transformation is fundamental to improve preventive strategies and to consequently prevent malignant transformation. The aim of this retrospective, observational, monocentric study is therefore the evaluation of the clinic/pathological prognostic factors associated with malignant transformation and recurrence of OPMDs after their primary surgical treatment.

## Materials and Methods

The database of patients affected by OPMDs and treated at the Department of Otolaryngology-Head and Neck Surgery of the University of Brescia, Italy, from January 1996 to June 2019 was reviewed. Data concerning survival were retrieved from mortality registries. Inclusion criteria were (a) first histopathological diagnosis of OPMD, treated with curative intent surgery (i.e., resection of the whole lesion in healthy margins), (b) availability of histological information regarding grade of dysplasia and margins of resection, and (c) availability of follow-up information regarding local recurrence either as dysplasia or carcinoma. Exclusion criteria were (a) previous surgery for OPMD performed elsewhere, (b) concurrent diagnosis of head and neck squamous cell carcinoma, and (c) previous history of OSCC within 2 years before the first diagnosis of OPMD.

Data management and study accomplishment are in accordance with principles stated in the Declaration of Helsinki; the study was approved by the local ethics committee (NP4713).

### Demographics, and Clinical and Pathological Data

Data concerning demographics, smoking, and alcohol habits (alcohol abuse has been considered as more than 4 alcohol units per day), Charlson Comorbidity Index (CCI) (10), and previous diagnosis of OSCC were collected. The first diagnosis of OPMDs has been recorded with the following characteristics: site; macroscopical aspect (leukoplakia or erythroplakia); grade of dysplasia according to the WHO classification into squamous intraepithelial neoplasia (SIN) 1, SIN2, SIN3; carcinoma *in situ* (CIS) (8); and margin status after surgery (resections with histological diagnosis of at least 1 mm of normal tissue from surgical margins were considered as negative margins; the presence of any grade of dysplasia at less than 1 mm from positive surgical margins was considered as positive).

### Statistical Analysis

Descriptive analysis was conducted expressing data in terms of median, inter-quartile range (IQR), range of values, and percentages. Differences in sex, age (cutoff: 65 years), high grade of dysplasia, and surgical margins of OPMD resection between patients with and without history of previous OSCC and according to the site of origin were assessed through Fisher’s exact test. Outcomes of interest of the survival analysis were (a) recurrence-free survival (RFS), defined as the time from surgery for OPMD to the diagnosis of OPMD relapse or carcinoma, whichever occurred first; (b) OPMD-specific RFS, defined as the time from surgery for OPMD to the diagnosis of OPMD relapse (recurrence in the form of carcinoma censored; new-onset OPMD distant from the site of primary OPMD surgically resected has been considered as OPMD relapse if within the same subsite of the primary); and (c) carcinoma-free survival (CFS), defined as the time from surgery for OPMD to malignant transformation (distant onset of OSCC from previous OPMDs has been considered as OPMD underwent malignant transformation if within the same subsite of the primary). A sub-analysis of CFS of patients without a previous history of OSCC was conducted as well. Survival curves with relative 95% confidence intervals (CIs) and the number of patients at risk by the time were plotted using the Kaplan–Meier method and compared with the log-rank test, reporting the 5-year survival estimates with relative 95% CI. Median follow-up time and corresponding IQR was estimated using the Kaplan–Meier estimator. Hazard ratio (HR) and 95% CI were derived using the Cox proportional hazard model. All the variables considered for univariate analysis were included in a multivariable model for RFS. Because of the low number of events (*N* = 26), multivariable analysis for CFS was limited to variables with univariate *p*-values <0.1.

Schoenfeld test was performed to assess the proportional hazards assumption. Statistical analysis was performed using R (version 4.1.2, R Foundation for Statistical Computing, Vienna, Austria); *p*-values < 0.05 (two-tailed) were considered statistically significant.

## Results

### Demographics and Clinical–Pathological Features

One-hundred-six patients with OPMDs met the inclusion criteria. The median age at first diagnosis was 64 years (IQR = 18.75). Sex was almost equally distributed (51.9% female patients). Alcohol abuse and current or previous smoking were reported by 4.7% and 31.1% of patients, respectively. A previous diagnosis of OSCC more than 2 years before index OPMD evaluation and treatment was documented in 31 cases (29.2%). A CCI > 3 was found in 14.1% of patients. Patients were primarily affected by OPMDs located on the tongue (43.3%). OPMDs have been classified as SIN1, SIN2, and SIN3-CIS in 46.2%, 30.2%, and 23.6% of cases, respectively. The main macroscopic aspect of OPMDs was leukoplakia (71.7%) followed by erythroplakia (16.0%) and leuko-erythroplakia (12.3%). All patients have been treated by surgical resection; margins were positive for the presence of dysplasia in 38.8% of cases. Further details are available in **Table 1**. Patients previously treated for OSCC showed a significantly higher risk of presenting with high-grade dysplasia (41.9%) than patients without previous OSCC (16%) (*p* = 0.009). No significant differences in sex (*p* = 0.859), age (cutoff: 65 years, *p* = 0.901) and OPMD surgical margins (*p* = 0.924) emerged according to history of previous OSCC. No differences in sex (*p* = 0.990), age (cutoff: 65 years, *p* = 0.843), high grade of dysplasia (*p* = 0.168), and margins of OPMD resection (*p* = 0.980) were observed according to subsite of origin.

**Table 1:** Descriptive analysis of the study population.

Patient number (n = 106)	Characteristics	Number (%)
**Sex**	Female	55 (51.9)
	Male	51 (48.1)
**Age at presentation (years)**	Mean 64.0	
	Range (30–94)	
**Smoke**	No	73 (68.9)
	Yes + former	33 (31.1)
**Alcohol abuse**	No	101 (95.3)
	Yes + former	5 (4.7)
**Charlson Comorbidity Index**	≥3	15 (14.1)
	<3	91 (85.9)
**Previous diagnosis of OSCC**	Yes	31 (29.2)
	No	75 (70.8)
**Previous diagnosis of cancer from any subsite (other than oral cavity)**	Yes	14 (13.2)
	No	92 (86.7)
**Subsite of primary OPMDs**	Tongue	46 (43.3)
	Cheek	35 (33.0)
	Hard Palate	12 (11.3)
	Alveolar Ridge	6 (5.6)
	Others	7 (6.8)
**Macroscopic Features of primary OPMDs**	Leukoplakia	76 (71.7)
	Erythroplakia	17 (16.0)
	Leuko-Erythroplakia	13 (12.3)
**Grading of primary OPMDs**	SIN1	49 (46.2)
	SIN2	32 (30.2)
	SIN3–CIS	25 (23.6)
**Margin assessed for the presence of dysplasia after surgical resection of primary OPMDs**	Positive	41 (38.8)
	Negative	65 (61.2)

OSCC, oral squamous cell carcinoma; SIN, squamous intraepithelial neoplasia; CIS, carcinoma in situ.

### Recurrence-Free Survival Analysis

During a median follow-up of 30.5 months (IQR = 44), 40 (37.7%) patients experienced a first local recurrence either as OPMD (22 cases, 20.7%) or OSCC (18 cases, 17.0%). Among the 22 patients with a first recurrence of OPMD, 9 (40.9%) experienced a further recurrence, and the rate of OSCC almost doubled (7, 31.8%). In total, malignant transformation has been assessed in 23.5% of cases with a median time to malignant transformation of 40 months (IQR = 57.5).

When disease recurred as OPMD, dysplasia was mostly of low grade (SIN1-2, 81.8%), with only 7.5% showing an increase in grade. Most of the relapse were in the same subsite of primary OPMDs (68.8% on tongue, 30.3% on cheek). All cases of dysplasia relapse were treated with surgery.

On the other side, when disease recurred as OSCC, 50% of patients had a previous diagnosis of OSCC. Tumor grading was low to intermediate in 52.0% of cases, staged as T1 in 39.1% and N0 in 47.8%. Surgical treatment of OSCC was performed in more than 90% of cases. Further data are presented in **Table 2**.

**Table 2:** Descriptive analysis of clinical evolution of OPMDs.

Patient number (*n* = 106)	Characteristics	Number (%)
**First Local Recurrence**	Yes	40 (37.7)
	OPMDs	22 (20.7)
	OSCC	18 (17.0)
	No	66 (62.3)
**Further Local Recurrence (among patients with first recurrence of OPMDs) (*n* = 22)**	Yes	9 (40.9)
	OPMDs	2 (9.0)
	OSCC	7 (31.8)
	No	13 (59.1)
**Overall OPMDs Recurrence**	Yes	24 (22.6)
	No	82 (77.4)
**Patients number (*n* = 24)**		
**OPMDs Recurrence Subsite**	Tongue	17 (68.8)
	Cheek	6 (30.3)
	Other	1 (0.9)
**OPMDs First Recurrence Grading**	SIN1/SIN2	20 (81.8)
	SIN3-CIS	4 (18.2)
**Overall Malignant Transformation**	Yes	25 (23.5)
	No	81 (76.0)
**Patient number (*n* = 25)**		
**OSCC Site**	Tongue	10 (40.0)
	Cheek	9 (36.0)
	Other	6 (24.0)
**OSCC Grading**	G1	9 (36.0)
	G2	12 (48.0)
	G3	4 (16.0)
**OSCC TNM (AJCC VIII edition)**		
** T**	T1	17 (68.0)
** N**	T>1	8 (21.0)
** M**	N0	21 (84.0)
	N>1	4 (16.0)
	M0	25 (100.0)
**OSCC Treatment**	Surgery	20 (80.0)
	Surgery + Radiotherapy	5 (20.0)

OPMDs, oral potentially malignant disorders; OSCC, oral squamous cell carcinoma; SIN, squamous intraepithelial neoplasia; CIS, carcinoma in situ; TNM, tumor-node-metastasis; AJCC, American Joint Committee of Cancer.

**Figure 1:**
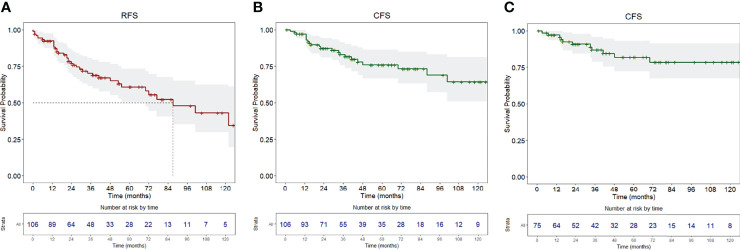
**(A)** Recurrence-free survival, **(B)** carcinoma-free survival of the whole population, **(C)** carcinoma-free survival of the population without a history of previous OSCC. Survival curves are reported with relative tables of patients at risk.

**Figure 2:**
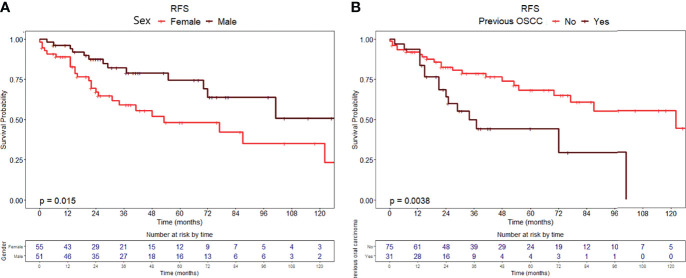
Recurrence-free survival curves with relative tables of patients at risk according to **(A)** sex and **(B)** history of previous OSCC.

RFS at 1, 5, and 10 years of follow-up was 92.4% (95% CI, 87.5–97.6%), 60.9% (95% CI, 50.5–73.4%), and 43.2% (95% CI, 29.8–62.5%), respectively (**Figure 1A**). Univariable analysis proved that female sex (HR = 2.24, 95% CI 1.15–4.38, *p* = 0.018) (**Figure 2A**) and a history of previous OSCC (HR = 2.60, 95% CI 1.33–5.07, *p* = 0.005) (**Figure 2B**), but not age at diagnosis (*p* = 0.403), site of origin (*p* = 0.584), grade of OPMD (*p* = 0.121), and margins of resection (*p* = 0.219), were associated with worse outcome. Both female (HR = 2.07, 95% CI 1.02–4.20, *p* = 0.044) and a history of previous OSCC (HR = 2.54, 95% CI 1.26–5.13, *p* = 0.009) were independently associated with relapse at the multivariable analysis (**Table 3**).

**Table 3:** Uni- and multi-variable analysis of the most relevant demographics and clinical–pathological factors according to RFS (recurrence-free survival) and CFS (carcinoma-free survival).

Variable		Recurrence-Free Survival				
		5-year RFS (95% CI)	Cox proportional-hazard model			
			Univariable analysis		Multivariable analysis	
			HR (95% CI)	*p*-value	HR (95% CI)	*p*-value
**Sex**	Male	74.3% (61.0–90.6%)	Reference	**0.018**	Reference	**0.044**
	Female	48.2% (34.5–67.6%)	2.24 (1.15–4.38)		2.07 (1.02–4.20)	
**Age at diagnosis, years**	<65	64.2% (50.5–81.6%)	Reference	0.403	Reference	0.889
	≥65	57.0% (42.4–76.7%)	1.31 (0.69–2.49)		1.05 (0.52–2.12)	
**Previous oral carcinoma**	No	68.3% (56.5–82.5%)	Reference	**0.005**	Reference	**0.009**
	Yes	44.3% (27.7–70.6%)	2.60 (1.33–5.07)		2.54 (1.26–5.13)	
**Site of origin**	Tongue	62.6% (48.4–80.9%)	Reference	0.584	Reference	0.498
	Other subsites	59.5% (44.8–79.1%)	1.20 (0.62–2.33)		1.26 (0.65–2.45)	
**Grade of dysplasia**	SIN1-2	63.9% (51.9–78.7%)	Reference	0.121	Reference	0.554
	SIN3/CIS	52.8% (35.7–78.1%)	1.70 (0.87–3.32)		1.25 (0.60–2.62)	
**Margins of resection**	Free	62.1% (48.8–78.9%)	Reference	0.219	Reference	0.360
	Involved by dysplasia	60.4% (45.8–79.6%)	1.50 (0.78–2.88)		1.37 (0.70–2.68)	

CI, confidence interval; CIS, carcinoma in situ; HR, hazard ratio; SIN, squamous intraepithelial neoplasia. The bold values are the statistically significant ones.

When considering OPMD-specific RFS, the only factor associated with higher risk was female sex (HR = 3.02, 95% CI 11.8–7.74, *p* = 0.021), whereas older age (*p* = 0.538), previous OSCC (*p* = 0.102), subsite (*p* = 0.408), grade of dysplasia (*p* = 0.947), and margins (*p* = 0.131) were not associated.

### Carcinoma-Free Survival Analysis

**Figure 3:**
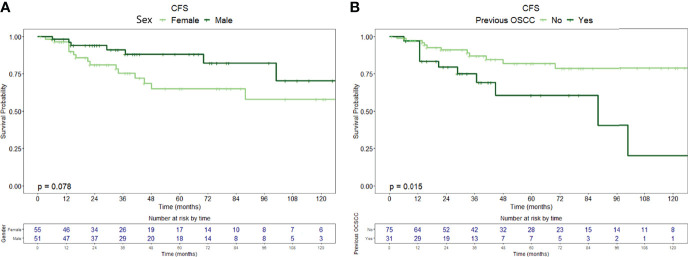
Carcinoma-free survival curves with relative tables of patients (whole population) at risk according to **(A)** sex and **(B)** history of previous OSCC.

Twenty-six patients (24.5%) were diagnosed with OSCC during follow-up, with a median time from diagnosis of OPMD to OSCC of 31 months (IQR = 51.2). In the whole population, CFS at 1, 5, and 10 years of follow-up was 97.1% (95% CI, 93.9–100%), 75.9% (95% CI, 66.7–86.5%), and 64.4% (95% CI, 51.0–81.3%), respectively (**Figure 1B**). At univariable analysis, age (*p* = 0.136), site of origin (*p* = 0.663), and positive margins (*p* = 0.479) did not influence CFS, whereas female sex (HR = 2.10, 95% CI 0.90–4.80, *p* = 0.085) (**Figure 3A**), grade of dysplasia (HR = 2.11, 95% CI 0.92–4.84, *p* = 0.077), and history of previous OSCC (HR = 2.65, 95% CI 1.18–5.96, *p* = 0.019) (**Figure 3B**) showed a significant or close-to-significant association. Multivariable analysis revealed the independent effect of previous OSCC (HR = 2.73, 95% CI 1.18–6.31, *p* = 0.019) (**Table 4**).

**Table 4:** Uni- and multi-variable analysis of the most relevant demographics and clinical–pathological factors according to CFS (carcinoma-free survival).

Variable		Carcinoma-Free Survival					Carcinoma-Free Survival (patients without previous OSCC)		
		5-year RFS (95% CI)	Cox proportional-hazard model					Cox proportional-hazard model	
			Univariable analysis		Multivariable analysis		5-year RFS (95% CI)	Univariable analysis	
			HR (95% CI)	*p*-value	HR (95% CI)	*p*-value		HR (95% CI)	*p*-value
**Sex**	Male	88.0% (78.4–98.7%)	Reference	*0.085*	Reference	*0.098*	97.1% (91.5–100%)	Reference	*0.063*
	Female	65.0% (51.1–82.6%)	2.10 (0.90–4.8)		2.05 (0.87–4.81)		68.3% (52.7–88.5%)	3.39 (0.93–12.3)	
**Age at diagnosis - years**	<65	85.8% (75.6–97.3%)	Reference	0.136			90.1% (79.8–100%)	Reference	0.163
	≥65	65.3% (50.8–83.8%)	1.85 (0.82–4.14)				73.2% (57.5–93.3%)	2.19 (0.73–6.62)	
**Previous oral carcinoma**	No	81.9% (72.1–93.2%)	Reference	**0.019**	Reference	**0.019**			
	Yes	60.6% (41.7–88.1%)	2.65 (1.18–5.96)		2.73 (1.18–6.31)				
**Site of origin**	Tongue	79.5% (67.6–93.4%)	Reference	0.663			92.6% (83.2–100%)	Reference	*0.066*
	Other subsites	70.6% (56.5–88.2%)	1.20 (0.52–2.78)				70.7% (54.5–91.7%)	4.25 (0.91–19.87)	
**Grade of dysplasia**	SIN1-2	79.4% (68.8–91.6%)	Reference	*0.077*	Reference	0.175	81.3% (69.9–94.4%)	Reference	0.758
	SIN3/CIS	66.5% (49.9–88.7%)	2.11 (0.92–4.84)		1.79 (0.77–4.16)		83.3% (64.7–100%)	1.22 (0.34–4.47)	
**Margins of resection**	Free	75.4% (63.4–89.2%)	Reference	0.479			82.9% (71.0–96.8%)	Reference	0.498
	Involved by dysplasia	78.1% (64.7–94.3%)	1.36 (0.58–3.15)				81.4% (66.3–100%)	1.51 (0.46–4.99)	

CI, confidence interval; CIS, carcinoma in situ; HR, hazard ratio; SIN, squamous intraepithelial neoplasia. The bold values are the statistically significant ones.

When restricting the analysis to patients with an OPMD without previous diagnosis of OSCC, we identified 75 patients. No demographic differences were found compared to the whole cohort (52% of female patients, median age at diagnosis of 64 years). OPMDs were evenly distributed throughout the oral cavity subsite (42.5% on the tongue, 60.3% on other subsites). SIN1, SIN2, and SIN3-CIS were diagnosed in 42 (56.0%), 21 (28.0%), and 12 cases (16.0%), respectively. Margins of resection were involved by dysplasia in 29 specimens (38.6%).

In this group of patients, a diagnosis of OSCC during follow-up was registered in 15 (20%), with a median time from diagnosis of OPMD to OSCC of 40 months (IQR = 57.5). CFS at 1, 5, and 10 years of follow-up was 97.2% (95% CI, 93.5–100%), 81.9% (95% CI, 72.1–93.2%), and 78.5% (95% CI, 67.4–91.5%), respectively (**Figure 1C**).

In univariable analysis, sex (HR = 3.39, 95% CI 0.93–12.30, *p* = 0.063) and OPMD location (HR = 4.25, 95% CI 0.91–19.87, *p* = 0.066) were close-to-significant. On the contrary, age at diagnosis (*p* = 0.150), grade of dysplasia (*p* = 0.760), and margin of resection (*p* = 0.500) were not significantly associated with an increased risk of malignant transformation (**Table 4**).

Considering the scarce number of events (*N* = 15) we did not perform a multivariable analysis.

## Discussion

Identification of high-risk OPMDs is an important unmet medical need (11). The main aim of our study was to evaluate the impact of clinical/histological factors related to patients primarily affected by OPMDs and the risk of recurrence and malignant transformation. In our cohort of patients surgically treated for OPMDs, we identified a malignant transformation rate of 24.5% with a median interval of 31 months (IQR = 51.2). When considering patients without a history of previous OSCC, malignant transformation rate was slightly lower (20%), with a longer median time from diagnosis of OPMD to OSCC (40 months, IQR = 57.5) and higher 5-year CFS (81.9% vs 75.9%).

Female sex and a previous diagnosis of OSCC were independent negative prognosticators for both RFS and CFS. We then analyzed the CFS in the subgroup of patients with an OPMD without any previous diagnosis of OSCC and we found that female sex and primarily location of OPMD outside the tongue were associated with shorter CFS.

There is no high consensus on risk factors in the natural history of OPMD. Other series reported that heavy tobacco smoking, alcohol consumption, immunosuppression status, and histological characteristics such as non-homogenous leukoplakia, OPMDs size >200 mm^2^, moderate or greater than moderate dysplasia, and lesions displaying a progression towards worse grades are at higher risk of malignant transformation (12, 13). Recently, a series evaluating high-grade OPMDs showed that factors significantly associated with higher risk of malignant transformation are age and positive margins after surgical excision (14). Differences in these factors could be explained by heterogeneity of inclusion criteria and possibly geographic risk factors. Moreover, it should be stressed that some series also considered patients with a previous diagnosis of OSCC within 2 years before diagnosis of OPMDs, when the risk of OSCC recurrence after curative treatment is higher (15), increasing the possibility to confound an OSCC recurrence with OPMD malignant transformation.

A higher incidence of malignant transformation for female patients is generally accepted in most of the series (16, 17). In this regard, a role of the sexual hormones could be hypothesized to contribute to malignant transformation. Oral carcinogenesis is a slowly multistep process, in which some genetic deregulations have been related to estrogen deficiency; a longer menopause period could pose women at higher risk of oral cancer, as some papers suggested (18, 19).

We have classified OPMDs according to the World Health Organization (WHO) classification (8), as SIN1, SIN2, SIN3, and CIS (20, 21). Coherently, we found an indication of a shorter CFS related to higher grade of dysplasia, though not statistically significant. The cutoff between SIN1, SIN2, or SIN3 is poorly defined and comparison between different series is difficult due to suboptimal inter-observer reliability and reproducibility. In this regard, data in the literature are not homogeneous. To overcome this, recently the WHO Classification of Head and Neck Tumors also tabled a binary system (high- versus low-grade dysplasia) and suggested cutoff criteria between the grades (22). Despite literature data showing higher risk of malignant transformation for lesions with high grade of dysplasia (2, 6, 12, 23), we could not confirm this in our series, indicating that classification of OPMDs dysplasia remains conflicting likely due to “subjective” definition.

According to recent review data (6), we found that OPMDs were mainly located on the tongue (43.3% of cases). The relationship between the site of OPMD and risk of malignant transformation is controversial (16, 24–27). In our study, we found that patients without a previous diagnosis of OSCC with lesions located outside the tongue had shorter CFS (HR = 4.25, *p* = 0.066). Lesions in the tongue are more easily identified by the patients themselves, with a consequent earlier diagnosis and less time to accumulate carcinogenetic alterations; this is more evident in patients without previous OSCC diagnosis and therefore undergoing less frequent follow-up visits.

A previous diagnosis of OSCC was an independent prognosticator of shorter RFS (HR = 2.54, *p* = 0.009) and CFS (HR = 2.73, 95% CI 1.18–6.31, *p* = 0.019). These data overlap with literature that showed how pre-existent molecular alteration could lead to an increased risk of malignant transformation. The loss of heterozygosity (LOH) has a central role on OPMDs’ malignant transformation. LOH on certain chromosomal loci such as 9p21 or 3p14 led to a higher risk of malignant transformation, due to implication on the encoding of various tumor suppressor genes, critical in malignant transformation, such as tumor protein 53 (TP53), and cyclin-dependent kinase inhibitor 2A (CDKN2A). Additional LOH on 17p or 8p or 11p or 4q or 13q could increase the risk of malignant transformation (28–30). An increased risk of OPMD recurrence and malignant transformation in patients with previous OSCC could be explained also by the theory of field cancerization referred to as the occurrence of molecular abnormalities in the tumor adjacent mucosal field, driven by the activities of local cancer stem cells (31).

Overall, the most relevant finding of our analysis is that clinical–pathological factors cannot thoroughly describe the risk of malignant transformation of OPMDs. Malignant transformation seems to be the sum of various risk factors, in this regard, the application of validated nomogram and the assistance of Deep Learning models could help in the identification of OPMDs at higher risk of malignant transformation (32, 33). With a window on the near future, we aim to get more precise molecular OPMD characterization to better define their malignant transformation risk and potentially tailor both therapeutic approach and the follow-up; moreover, a better characterization could allow patient enrichment for prevention trials.

Intriguingly, it is possible to describe, from a transcriptomic point of view, six distinct clusters of disease based on main biological features and deregulated pathways, mirroring the pathways present in head and neck squamous cell carcinomas: defense response, immunoreactive, human papillomavirus-related, classical, hypoxia, and mesenchymal clusters, where mesenchymal, hypoxia, and classical clusters have a higher risk of malignant transformation if compared with immunoreactive clusters (34). Even immune infiltration seems to play an important role in malignant transformation for OPMDs (35); consequently, ongoing clinical trials are exploring the activity of ICIs such as avelumab (NCT04504552) and sintilimab (NCT04065737) in high-risk OPMDs defined based on molecular criteria (i.e., LOH 3p14 and/or 9p21), and pembrolizumab (NCT03603223) in OPMDs.

The main limitations of our cohort were the retrospective nature of the study, the lack of information on previous OPMD before OSCC, and the absence of more in-depth histological (e.g., immune infiltration and HPV status) analysis.

## Conclusions

In our study, only a previous OSCC was a prognosticator of further OSCC recurrence. Molecular characterization of OPMDs should be included in future studies, better stratify the risk of malignant transformation, and consequently define an enriched population for preventative strategies within clinical trials.

## Data Availability Statement

The datasets presented in this study can be found in online repositories. The names of the repository/repositories and accession number(s) can be found at: luigilorini91@gmail.com.

## Author Contributions

LL, PB, and MT conceived the study. LL, MT, PB, and CM contributed to manuscript writing. PB, CP, DM, AD, FF, AP, AG, AM, EB, CR, DL, and MR revised the manuscript. SC and MT contributed to the statistical analysis. CM, LL, MT, MF, SB, AO, AB, and CG collected data. All authors contributed to the article and approved the submitted version.

## Funding

This work has been funded by Associazione Italiana Ricerca sul Cancro (AIRC), thanks to an Investigator Grant (IG 21740 to PB).

## Conflict of Interest

PB declares advisory board participation or conference honoraria from Merck, Sanofi-Regeneron, Merck Sharp & Dohme, Sun Pharma, Angelini, Molteni, Bristol-Myers Squibb, GSK, and Nestlè.

The remaining authors declare that the research was conducted in the absence of any commercial or financial relationships that could be construed as a potential conflict of interest.

## Publisher’s Note

All claims expressed in this article are solely those of the authors and do not necessarily represent those of their affiliated organizations, or those of the publisher, the editors and the reviewers. Any product that may be evaluated in this article, or claim that may be made by its manufacturer, is not guaranteed or endorsed by the publisher.
